# Supplementary material for: HIVprotI: an integrated web based platform for prediction and design of HIV proteins inhibitors
Source: J Cheminform. 2018 Mar 9;10:12. doi: 10.1186/s13321-018-0266-y (PMC5845081; doi:10.1186/s13321-018-0266-y)
Supplement: Supplementary file 2 — Additional file 2. Source code of HIVProtI web server. [file 13321_2018_266_MOESM2_ESM.zip › HIVprotI_Source-code/hivprotI/weka-3-6-6/doc/weka/core/neighboursearch/balltrees/MedianOfWidestDimension.html]

MedianOfWidestDimension


---


|  |  |  |
| --- | --- | --- |
| **Overview** \| **Package** \| **Class** \| **Tree** \| **Deprecated** \| **Index** \| **Help** \| **Weka's home** | |  |
| **PREV CLASS**   **NEXT CLASS** | **FRAMES**    **NO FRAMES**     **All Classes** |  |
| SUMMARY: NESTED \| FIELD \| CONSTR \| METHOD | DETAIL: FIELD \| CONSTR \| METHOD |  |


---


## weka.core.neighboursearch.balltrees Class MedianOfWidestDimension

```
java.lang.Object
  weka.core.neighboursearch.balltrees.BallSplitter
      weka.core.neighboursearch.balltrees.MedianOfWidestDimension
```

**All Implemented Interfaces:**: java.io.Serializable, OptionHandler, RevisionHandler, TechnicalInformationHandler

---

``` public class MedianOfWidestDimension extends BallSplitter implements OptionHandler, TechnicalInformationHandler ```

Class that splits a BallNode of a ball tree based on the median value of the widest dimension of the points in the ball. It essentially implements Omohundro's KD construction algorithm.


BibTeX:

```
 @techreport{Omohundro1989,
    author = {Stephen M. Omohundro},
    institution = {International Computer Science Institute},
    month = {December},
    number = {TR-89-063},
    title = {Five Balltree Construction Algorithms},
    year = {1989}
 }
```


Valid options are:

```
 -N
  Normalize dimensions' widths.
```

**Version:**
:   $Revision: 1.2 $

**Author:**
:   Ashraf M. Kibriya (amk14[at-the-rate]cs[dot]waikato[dot]ac[dot]nz)

**See Also:**: Serialized Form

---

**Constructor Summary**

`MedianOfWidestDimension()`
  
          Constructor.

`MedianOfWidestDimension(int[] instList,
Instances insts,
EuclideanDistance e)`
  
          Constructor.


| **Method Summary** | |
| --- | --- |
| `boolean` | `getNormalizeDimWidths()`             Whether we are normalizing the widths(ranges) of the dimensions (attributes) or not. |
| `java.lang.String[]` | `getOptions()`             Gets the current settings. |
| `java.lang.String` | `getRevision()`             Returns the revision string. |
| `TechnicalInformation` | `getTechnicalInformation()`             Returns an instance of a TechnicalInformation object, containing detailed information about the technical background of this class, e.g., paper reference or book this class is based on. |
| `java.lang.String` | `globalInfo()`             Returns a string describing this nearest neighbour search algorithm. |
| `java.util.Enumeration` | `listOptions()`             Returns an enumeration describing the available options. |
| `java.lang.String` | `normalizeDimWidthsTipText()`             Returns the tip text for this property. |
| `int` | `select(int attIdx, int[] indices, int left, int right, int k)`             Implements computation of the kth-smallest element according to Manber's "Introduction to Algorithms". |
| `void` | `setNormalizeDimWidths(boolean normalize)`             Should we normalize the widths(ranges) of the dimensions (attributes) before selecting the widest one. |
| `void` | `setOptions(java.lang.String[] options)`             Parses a given list of options. |
| `void` | `splitNode(BallNode node, int numNodesCreated)`             Splits a ball into two. |

**Methods inherited from class weka.core.neighboursearch.balltrees.BallSplitter**

`setEuclideanDistanceFunction, setInstanceList, setInstances`

**Methods inherited from class java.lang.Object**

`equals, getClass, hashCode, notify, notifyAll, toString, wait, wait, wait`

**Constructor Detail**

### MedianOfWidestDimension

```
public MedianOfWidestDimension()
```

:   Constructor.

---


### MedianOfWidestDimension

```
public MedianOfWidestDimension(int[] instList,
                               Instances insts,
                               EuclideanDistance e)
```

:   Constructor.

    **Parameters:**: `instList` - The master index array.: `insts` - The instances on which the tree is (or is to be) built.: `e` - The Euclidean distance function to use for splitting.


**Method Detail**

### globalInfo

```
public java.lang.String globalInfo()
```

:   Returns a string describing this nearest neighbour search algorithm.

    :   **Returns:**: a description of the algorithm for displaying in the explorer/experimenter gui

---


### getTechnicalInformation

```
public TechnicalInformation getTechnicalInformation()
```

:   Returns an instance of a TechnicalInformation object, containing detailed
    information about the technical background of this class, e.g., paper
    reference or book this class is based on.

    :   **Specified by:**: `getTechnicalInformation` in interface `TechnicalInformationHandler`
    :   **Returns:**: the technical information about this class

---


### splitNode

```
public void splitNode(BallNode node,
                      int numNodesCreated)
               throws java.lang.Exception
```

:   Splits a ball into two.

    :   **Specified by:**: `splitNode` in class `BallSplitter`
    :   **Parameters:**: `node` - The node to split.: `numNodesCreated` - The number of nodes that so far have been created for the tree, so that the newly created nodes are assigned correct/meaningful node numbers/ids. **Throws:**: `java.lang.Exception` - If there is some problem in splitting the given node.

---


### select

```
public int select(int attIdx,
                  int[] indices,
                  int left,
                  int right,
                  int k)
```

:   Implements computation of the kth-smallest element according
    to Manber's "Introduction to Algorithms".

    :   **Parameters:**: `attIdx` - The dimension/attribute of the instances in which to find the kth-smallest element.: `indices` - The master index array containing indices of the instances.: `left` - The begining index of the portion of the master index array in which to find the kth-smallest element.: `right` - The end index of the portion of the master index array in which to find the kth-smallest element.: `k` - The value of k **Returns:**: The index of the kth-smallest element

---


### normalizeDimWidthsTipText

```
public java.lang.String normalizeDimWidthsTipText()
```

:   Returns the tip text for this property.

    :   **Returns:**: tip text for this property suitable for displaying in the explorer/experimenter gui

---


### setNormalizeDimWidths

```
public void setNormalizeDimWidths(boolean normalize)
```

:   Should we normalize the widths(ranges) of the dimensions (attributes)
    before selecting the widest one.

    :   **Parameters:**: `normalize` - Should be true if the widths are to be normalized.

---


### getNormalizeDimWidths

```
public boolean getNormalizeDimWidths()
```

:   Whether we are normalizing the widths(ranges) of the dimensions (attributes)
    or not.

    :   **Returns:**: true if widths are being normalized.

---


### listOptions

```
public java.util.Enumeration listOptions()
```

:   Returns an enumeration describing the available options.

    :   **Specified by:**: `listOptions` in interface `OptionHandler` **Overrides:**: `listOptions` in class `BallSplitter`
    :   **Returns:**: an enumeration of all the available options.

---


### setOptions

```
public void setOptions(java.lang.String[] options)
                throws java.lang.Exception
```

:   Parses a given list of options.
    Valid options are:

    ```
     -N
      Normalize dimensions' widths.
    ```

    :   **Specified by:**: `setOptions` in interface `OptionHandler` **Overrides:**: `setOptions` in class `BallSplitter`
    :   **Parameters:**: `options` - the list of options as an array of strings **Throws:**: `java.lang.Exception` - if an option is not supported

---


### getOptions

```
public java.lang.String[] getOptions()
```

:   Gets the current settings.

    :   **Specified by:**: `getOptions` in interface `OptionHandler` **Overrides:**: `getOptions` in class `BallSplitter`
    :   **Returns:**: An array of strings suitable for passing to setOptions or to be displayed by a GenericObjectEditor.

---


### getRevision

```
public java.lang.String getRevision()
```

:   Returns the revision string.

    :   **Specified by:**: `getRevision` in interface `RevisionHandler` **Overrides:**: `getRevision` in class `BallSplitter`
    :   **Returns:**: the revision


---


|  |  |  |
| --- | --- | --- |
| **Overview** \| **Package** \| **Class** \| **Tree** \| **Deprecated** \| **Index** \| **Help** \| **Weka's home** | |  |
| **PREV CLASS**   **NEXT CLASS** | **FRAMES**    **NO FRAMES**     **All Classes** |  |
| SUMMARY: NESTED \| FIELD \| CONSTR \| METHOD | DETAIL: FIELD \| CONSTR \| METHOD |  |


---
